# Neuropeptide Y-Positive Neurons in the Dorsomedial Hypothalamus Are Involved in the Anorexic Effect of Angptl8

**DOI:** 10.3389/fnmol.2018.00451

**Published:** 2018-12-18

**Authors:** Rui Wang, Junhua Yuan, Caishun Zhang, Liuxin Wang, Yuan Liu, Limin Song, Weizhen Zhong, Xi Chen, Jing Dong

**Affiliations:** ^1^Department of Special Medicine, Medical College, Qingdao University, Qingdao, China; ^2^Institute of Foundation Medicine, Medical College, Qingdao University, Qingdao, China; ^3^Department of Physiology, Medical College, Qingdao University, Qingdao, China

**Keywords:** Angptl8, neuropeptide Y, appetite, dorsomedial hypothalamus, adipose tissue

## Abstract

Angiopoietin-like protein 8 (Angptl8), a recently identified member of the angiopoietin-like protein family (ANGPTLs), is a 22-kDa peptide synthesized in the liver. It participates in lipid metabolism by inhibiting lipoprotein lipase (LPL) activity, consequently increasing the triglyceride levels. Despite evidence that Angptl8 is involved in feeding control, the underlying mechanisms are unclear. Central and peripheral injections of Angptl8 significantly decreased food intake. Angptl8 was widely expressed in appetite-related nuclei, including the paraventricular nucleus (PVN), the dorsomedial hypothalamus (DMH), the ventromedial hypothalamus, and the arcuate nucleus (ARC) in the hypothalamus. Peripheral Angptl8 administration decreased c-Fos-positive neurons in the DMH. Central Angptl8 administration decreased c-Fos-positive neurons in the DMH and PVN but increased these neurons in the ARC. Angptl8 inhibited appetite via neuropeptide Y (NPY) neurons in the DMH. Furthermore, the chronic administration of Angptl8 decreased body weight gain and altered adipose tissue deposits. Nevertheless, neither peripheral nor central Angptl8 influenced the brown adipose tissue (BAT) morphology or uncoupling protein 1 (Ucp-1) expression in BAT. Taken together, these data suggested that Angptl8 modulates appetite and energy homeostasis.

## Introduction

Angiopoietin-like protein 8 (Angptl8) is a 22-kDa peptide synthesized in the liver (Ren et al., 2012). Although the receptor of Angptl8 has not been discovered, it is widely distributed in white adipose tissue (WAT), BAT, and the brain (Quagliarini et al., 2012). Angptl8 inhibits the activity of LPL, consequently increasing triglyceride levels and decreasing FFA levels in the serum (Zhang, 2012; Wang et al., 2013; Haller et al., 2017).

Plasma Angptl8 levels are significantly elevated in anorexic patients and are reduced in morbidly obese subjects (Barja-Fernández et al., 2015). In addition, fasting suppresses Angptl8 expression in the liver, while refeeding increases its expression levels (Quagliarini et al., 2012; Zhang and Abou-Samra, 2013), suggesting that circulating Angptl8 is involved in the feeding control. Therefore, we evaluated the effects of exogenous Angptl8 on appetite regulation. As Angptl8 is distributed in both peripheral tissues and the brain (Quagliarini et al., 2012), we hypothesized that Angptl8 has different effects on energy homeostasis in the peripheral and central systems, like Angptl3 (Kim et al., 2015); accordingly, the effects of peripheral and central administration were both examined.

The hypothalamus contains several nuclei involved in the central control of appetite, including the ARC, the PVN, the VMH, and the dorsomedial hypothalamus (DMH) (Palkovits, 2003). Although Quagliarini et al. (2012) demonstrated the expression of Angptl8 in the brain, its distribution in these nuclei related to appetite regulation is unclear and the further investigation are needed to determine whether exogenous Angptl8 could influence the activity of specific type of neurons.

Neuropeptide Y (NPY) is an important hypothalamic orexigenic peptide (Clark et al., 1984). NPY-ergic neurons of the hypothalamic ARC project to the PVN and DMH, stimulating feeding and inhibiting thermogenesis (Baker and Herkenham, 1995; Wang et al., 1997). Central NPY-positive neurons are involved in the modulation of feeding and body weight (Meister, 2007), fat formation (Zhang et al., 2014), and Ucp-1 expression (Bi and Li, 2013). Ucp-1 is expressed in the inner mitochondrial membrane of brown adipocytes, and plays an important role in heat production (Abegg et al., 2016).

In the present study, we measured the expression of Angptl8 in the hypothalamus, explored whether exogenous Angptl8 regulates appetite and energy homeostasis, and further explored its potential mechanisms.

## Materials and Methods

### Animals

Male C57BL/6J mice (8–10 weeks) were purchased from Qingdao Institute of Drug Control and were raised in standard housing conditions (22–24°C, illumination from 6:00 to 18:00) on a chow diet for 7 days. This study was performed in accordance with the recommendations of the Animal Welfare Officers at Medical College of Qingdao University. The protocols were approved by the Qingdao University Animal Care and Use Committee in keeping with the National Institutes of Health guidelines.

### Experiment 1: Angptl8 Expression in the Hypothalamus

To measure the central expression of Angptl8, animals on chow diet and food deprivation for 12 h were anesthetized with chloral hydrate (400 mg/kg/ip) and ketorolac (1 mg/kg/im; pain killer), and were then perfused at 9:00 AM with normal saline (NS) and 4% paraformaldehyde. The animals were decapitated; the brains were carefully dissected, rinsed with cold NS, and fixed in 4% paraformaldehyde for 24 h.

### Experiment 2: Assessment for Effects of Peripheral Angptl8

To measure the peripheral effects of Angptl8, mice were injected with 0.1 ml of NS and a low concentration (3 μg/ml) or high concentration (30 μg/ml) of human recombinant Angptl8 (051-61, Phoenix Pharmaceuticals, Inc., St. Joseph, MO, United States) via tail veins before the end of the light cycle (18:00), as described in our previous study (Dong et al., 2013).

For acute effects, following 12-h food deprivation, Angptl8 was injected into mice. Then, 12-h food intake after refeeding was measured using an electronic scale (TE412-L; Sartorius, Göttingen, Germany). All the remaining food including the spillage was weight every hour and after each measurement we supplied the mice with new and intact food. Animals used for immunostaining were sacrificed 2 h after the injection.

To investigate the effects of chronic peripheral Angptl8 administration, the body weights of mice were measured before the injections and mice were treated intravenously with NS or high-concentration Angptl8 daily (18:00) for 9 days. On day 9, animals were sacrificed 2 h after the injection. Blood samples were obtained from the orbital sinus and were stored at -20°C, while other samples, including the BAT from the interscapular region, epididymal adipose tissue and inguinal adipose tissue, were weighed and stored at-80°C. The ratio of fat to body weight was calculated.

### Experiment 3: Assessment for Effects of Central Angptl8

To measure the central effects of Angptl8, mice were randomly divided into three groups: NS, low concentration (2 μl, 0.03 μg/μl) and high concentration (2 μl, 0.3 μg/μl) groups. Before the injection, a cannula was implanted into the brain for administration. Mice were anesthetized with chloral hydrate (400 mg/kg/ip) and ketorolac (1 mg/kg/im; pain killer) and then were positioned in a stereotaxic apparatus (68526; RWD Life Science, Shenzhen, China). Cannulae (26 gauge; RWD Life Science, China) were implanted into the lateral ventricles (2.2–2.3 mm depth, 1 mm caudal to bregma, 0.3 mm lateral from the sagittal suture) as described previously (Bellocchio et al., 2013). The cannulae were fixed with dental cement and a stainless-steel screws. A 7-day recovery period followed. The timepoint of treatment, perimeter measurement, and tissue collection were the same as those in Experiment 2. At the end of the study, the mice were sacrificed and cannula placement was verified by the injection of 1 μl of Pontamine Sky Blue. Data with correct injection sites were included in the analyses.

### Histological Assessment

#### HE-Staining

Partial BAT was fixed in 4% formaldehyde in PB (pH 7.4) for 24 h. After dehydration with various concentrations of ethanol and clarifying with xylene, BATs were embedded in mixed paraffin prior to sectioning. BATs were cut into 5-μm sections using a paraffin slicing machine (RM2016; Leica, Wetzlar, Germany). Sections were stained using a Hematoxylin-Eosin Staining Kit (G1120; Solarbio, Beijing, China). Cell numbers were analyzed using ImagePro-Plus software.

#### Immunohistochemistry and Immunofluorescence

Mice brains were fixed with 4% paraformaldehyde overnight and were transferred into 30% sucrose at 4°C for 12 h. The brains were embedded in Optimal Cutting Temperature (OCT) Compound (4583; Sakura, Torrance, CA, United States). OCT-embedded brains were cut into 15-μm sections using a microtome (CM1860; Leica) and sections cut from the same nuclei position of each animal were put on the glass slide and roasted for 4 h at 60°C in an incubator (DHG-9101; Sanfa, Yangzhou, China). After antigen retrieval, sections were blocked with 1% fetal bovine serum (FBS, 1213G057; Solarbio) dissolved in phosphate-buffered saline.

After blocking with bovine serum albumin for 2 h at room temperature, sections for immunohistochemistry were incubated with a c-Fos primary antibody (rabbit anti mouse, ab190289, 1:2000; Abcam, Cambridge, United Kingdom) for 2 h and HRP (horse radish peroxidase) secondary antibody (PV-6001; Zsbio, Tianjin, China) for 20 min at 37°C. They were then stained using a DAB Kit (ZLI-9018; Zsbio). Morphology was assessed using a light microscope (CX31; Olympus, Tokyo, Japan).

After they are blocked with bovine serum, sections were incubated with primary antibodies overnight at 4°C and incubated with secondary antibodies for 2 h at room temperature. Primary antibodies were as follows: Angptl8 (rabbit anti mouse, ab180915, 1:200; Abcam), c-Fos (rabbit anti mouse, ab190289, 1:2000; Abcam) (Lin et al., 2017), and NPY (sheep anti mouse, ab6173, 1:100; Abcam,). Goat anti-rabbit secondary antibody (ZF-0311, 1:100; Zsbio) and donkey anti-sheep secondary antibody (NL101, 1:200; R&D Systems, Minneapolis, MN, United States) were used. The fluorescence intensity was evaluated using a fluorescence microscope (Axio Observer A1; Zeiss, Oberkochen, Germany).

### Western Blotting

Brown adipose tissues were lysed in proteinase inhibitors in RIPA buffer, followed by centrifugingation at 4°C for 15 min at 12,000 *g*. Supernatants were collected and the BCA Protein Assay Kit (P0012; Beyotime, Shanghai, China) and a microplate reader (M5; MD-SpectraMax, Molecular Devices, San Jose, CA, United States) was used to determine total protein concentrations according to the manufacturers’ instructions. Then 50 μl of total protein from the supernatants and added was supplemented with the corresponding volume of 5× SDS loading buffer and lysis buffer to obtain a final protein concentration of 35 μg/10 μl, followed by boiling for 5 min. The same amount of protein was subjected to SDS-PAGE, followed by transfer to polyvinylidene fluoride membranes (PVDF, IPVH00010: Millipore, Burlington, MA, United States), which were activated by methanol. Membranes were blocked with 5% FBS for 2 h at room temperature and incubated with corresponding primary antibodies at 4°C overnight and with secondary antibodies at room temperature for 1 h. The protein bands were developed by using Immobilon Western Chemiluminescent Substrate (Millipore, cat. no. WBKLS0100, 200 μl). The primary antibodies were Ucp-1 (ab10983, 1:2000; Abcam) and β-actin (#4967, 1:4000; CST, Danvers, MA, United States). Secondary antibody goat anti-rabbit IgG H&L (HRP) (ab6721, Abcam, 1:2000) is used. Image J was used to analyze intensity, as described in our previous study (Yuan et al., 2017).

### Real-Time PCR

Total RNAs from the BAT or WAT were extracted using TRIzol reagents (135306; Ambion, Foster City, CA, United States). Then 4× gDNA Wiper Mix was used to rinse genomic DNA. Subsequently, the mRNAs were reverse-transcribed into cDNA by using 5× HisScript II qRT SuperMix II according to the manufacturer’s protocol. Relative mRNA levels were determined using the SYBR Green RT-PCR Kit (Q311; Vazyme Biotech Co., Ltd., Nanjing, China) and the Realplex Real Time PCR Thermocycle Instrument (Realplex 4; Eppendorf, Westbury, NY, United States). Real-time PCR data were analyzed with the comparative CT method. β-actin and GAPDH mRNA levels were used as an internal control. Primers used in this study for the qPCR analysis were as followed: *β-actin*: 5′-AGGCCCAGAGCAAGAGAGGTA-3′, 5′-GGGGTGTTGAAGGTCTCAAACA-3′; *GAPDH*: 5′-ACAGTCCATGCCATCACTGC-3′, 5′-GATCCACGACGGACACATTG-3′ (Ruigroka et al., 2018); *Ucp-1*: 5′-ACTGCCACACCTCCAGTCATT-3′, 5′-CTTTGCCTCACTCAGGATTGG-3′ (Zhou et al., 2014).

### Measurement of FFA Concentrations in the Plasma

The Cu-colorimetric method using a Non-esterified Fatty Acid assay kit (BC0595; Solarbio, China) was used to estimate the plasma FFA levels. The results are expressed as absorbance values.

### Statistical Analysis

Data are presented as means ± standard error of the means (SEMs). Value was regarded as a potential outlier if it deviated two times of SEM from the mean and undergone further test. We performed statistical analysis before and after deletion of the potential outlier. And if the results were inconsistent, the value would be categorized as an outlier and deleted. Statistical analyses were performed using a commercially available statistical package (SPSS 17.0). Comparisons between two groups were analyzed using the Student’s *t*-test. One-way ANOVA followed by a *post hoc* least significance difference test was used for comparisons among three groups. LSD (Least significance difference) test was used as *post hoc* test. *P*-value <0.05 was considered statistically significant.

## Results

### Peripheral and Central Angptl8 Decreased Food Intake and 24 h Body Weight

**FIGURE 1:**
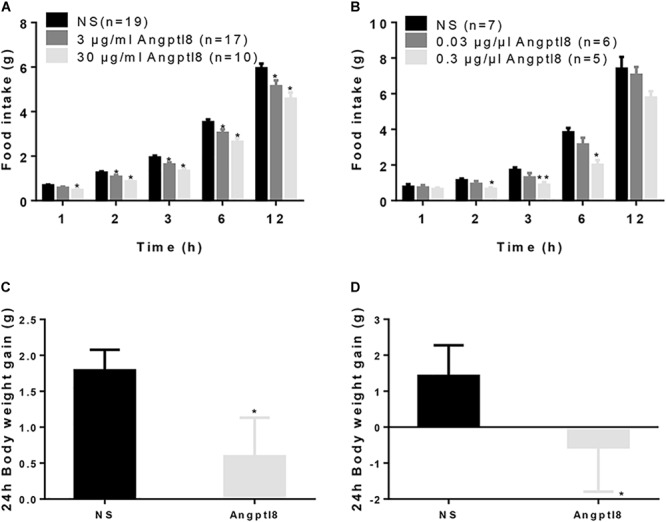
Effect of peripheral and central Angptl8 on food intake and 24 h body weight. **(A)** After 12-h food deprivation, the I.V. injection of Angptl8 (0.1 ml) decreased nocturnal cumulative food intake from 1 to 12 h after injection. **(B)** After 12-h food deprivation, I.C.V. injection of Angptl8 (2 μl) decreased nocturnal cumulative food intake from 2 to 6 h after injection. **(C)** Peripheral Angptl8 (0.1 ml; 30 μg/ml) decreased 24-h body weight gain (*n* = 8). **(D)** Central Angptl8 for (2 μl; 0.3 μg/μl) decreased 24-h body weight gain (*n* = 6). Data are means ± SEM. ^∗^*P* < 0.05 and ^∗∗^*P* < 0.05 relative to the NS control.

After 12-h food deprivation, Angptl8 (3 μg/ml or 30 μg/ml) was injected via the I.V. route at the beginning of the dark phase and the cumulative food intake overnight was measured. Angptl8 induced a concentration-dependent decrease in food intake beginning at 2 h (low concentration: 1.07 ± 0.83 g vs. 1.26 ± 0.06 g, high concentration: 0.86 ± 0.08 g vs. 1.26 ± 0.06 g, *P* < 0.05; Figure 1A) and this decrease was sustained for 12 h (low concentration: 5.14 ± 0.26 g vs. 5.95 ± 0.21 g, high concentration: 4.58 ± 0.28 g vs. 5.95 ± 0.21 g, *P* < 0.05; Figure 1A) after the injection. We found that, compared with NS-treated counterparts, I.C.V. microinjections of Angptl8 (0.3 μg/μl) significantly reduced nocturnal cumulative food intake beginning at 2 h (0.65 ± 0.08 vs. 1.14 ± 0.09 g, *P* < 0.05; Figure 1B), which was sustained until 6 h (2.00 ± 0.28 vs. 3.82 ± 0.25 g, *P* < 0.05; Figure 1B). In addition, the I.V. administration of Angptl8 (30 μg/ml) (1.79 ± 0.29g vs. 0.594 ± 0.539g, *P* < 0.05; Figure 1C) or central Angptl8 (0.3 μg/μl) (1.42 ± 0.85 g vs. -0.56 ± 1.23 mmol/L, *P* < 0.05; Figure 1D) resulted in significantly lower 24-h body weight gain than that in the NS group.

### Angptl8 Was Expressed in Appetite-Related Nuclei in the Hypothalamus

**FIGURE 2:**
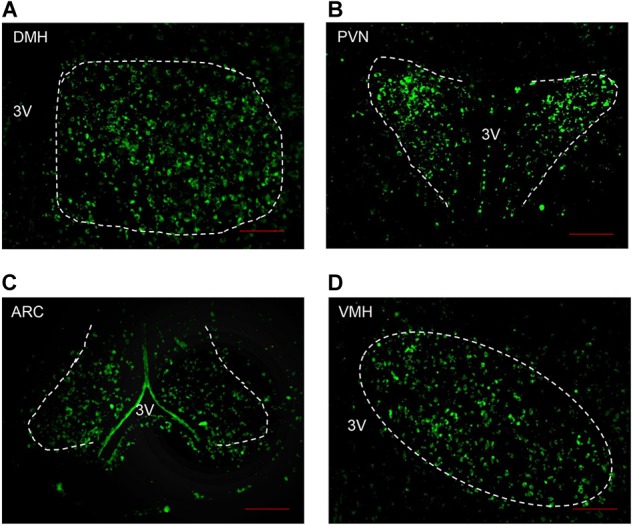
Angptl8 expression in appetite-related nuclei in the hypothalamus. Angptl8 was expressed in the hypothalamic PVN **(B)**, DMH **(A)**, ARC **(C)**, and VMH **(D)** in normal mice on chow diet. Scale bar = 200 μm.

As Angptl8 is expressed in the brain (Quagliarini et al., 2012) and influences cumulative food intake, we measured the relationship between Angptl8 and the feeding regulating center in the hypothalamus. The brains of mice on chow diets were harvested. We found that Angptl8 is abundantly expressed in the DMH, PVN, ARC, and VMH in the hypothalamus (Figures 2A–D). Angptl8 expression was qualified in appetite related nucleus in feeding and food deprivation state, but no significant difference was found (Supplementary Figure S1).

### Peripheral Angptl8 Decreased c-Fos-Positive Neurons Expression in the DMH

**FIGURE 3:**
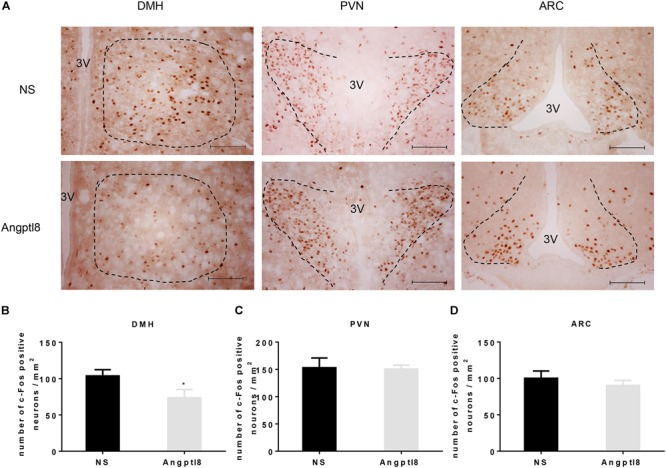
Peripheral Angptl8 decreased c-Fos-positive neurons expression in the DMH of the hypothalamus. **(A)** c-Fos-positive neurons change in hypothalamus 2 h after tail intravenous injection with NS and Angptl8. **(B–D)** The numbers of c-Fos-immunopositive cells in the DMH (*n* = 8), PVN (*n* = 6), and ARC (*n* = 8) are expressed as means ± SEM. ^∗^*P* < 0.05 relative to the NS control group. Scale bar in *A* = 100 μm.

To further study whether Angptl8 modulated the food intake by influencing the activity of neurons in the hypothalamus, after 12-h food deprivation, c-Fos-positive neurons in various hypothalamic nuclei were counted after the I.V. injection of 0.1 ml of Angptl8 (30 μg/ml) (Figure 3A). Compared with the control group, activated neurons in the Angptl8 group were decreased only in the DMH (103.70 ± 8.55 vs. 73.50 ± 11.52 cells per section in NS and Angptl8 treated animals, respectively; *P* < 0.05; Figure 3B). A decrease in c-Fos-positive neurons expression was observed, but there was no significant difference between groups in the PVN (153.00 ± 17.89 vs. 150.50 ± 7.24 cells per section in NS and Angptl8 treated animals, respectively; *P* > 0.05; Figure 3C) and ARC (100.13 ± 10.03 vs. 90.25 ± 7.12 cells per section in NS and Angptl8 treated animals, respectively; *P* > 0.05; Figure 3D).

### Central Angptl8 Injection Influenced c-Fos-Positive Neuron Expression in the DMH, PVN, and ARC

**FIGURE 4:**
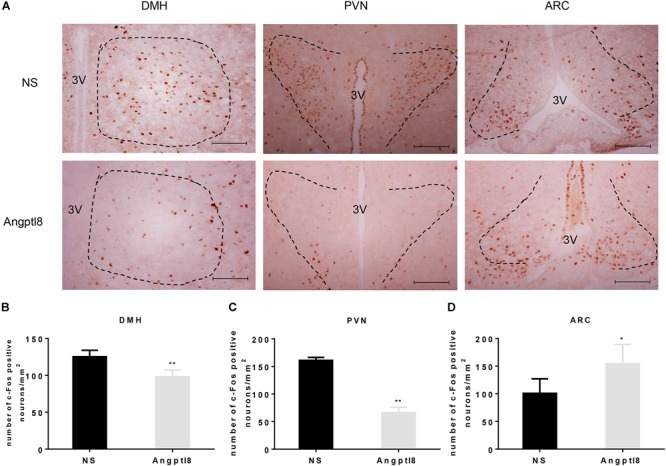
Central Angptl8 influenced c-Fos-positive neurons expression in the PVN, DMH, and ARC. **(A)** Changes in c-Fos-positive neurons in the hypothalamus 2 h after I.C.V. injection with NS and Angptl8. **(B–D)** The numbers of c-Fos immunoreactive cells in the DMH (*n* = 5), PVN (*n* = 3), and ARC (*n* = 6) are expressed as means ± SEM. ^∗^*P* < 0.05, ^∗∗^*P* < 0.001 relative to the NS control group. Scale bar in *A* = 100 μm.

The central injection of Angptl8 reduced neuron activation (Figure 4A) in the DMH (125.13 ± 8.86 vs. 98.00 ± 9.02 cells per section in NS and Angptl8 treated animals, respectively; *P* < 0.05; Figure 4B), as previously observed after the I.V. injection of Angptl8 or NS. In addition, this reduction in c-Fos-positive nuclei was clearly detected in the PVN (161.00 ± 5.41 vs. 66.33 ± 9.65 cells per section in NS and Angptl8 treated animals, respectively; *P* < 0.05; Figure 4C), but c-Fos-positive nuclei were increased in the ARC (100.80 ± 26.26 vs. 154.08 ± 35.15 cells per section in NS and Angptl8 treated animals, respectively; *P* < 0.05; Figure 4D). These results suggested that Angptl8 influences food intake via the DMH, PVN, and ARC nuclei.

### Double Staining of c-Fos and NPY in the Hypothalamic PVN, DMH, and ARC After the I.C.V. Injection of Angptl8

**FIGURE 5:**
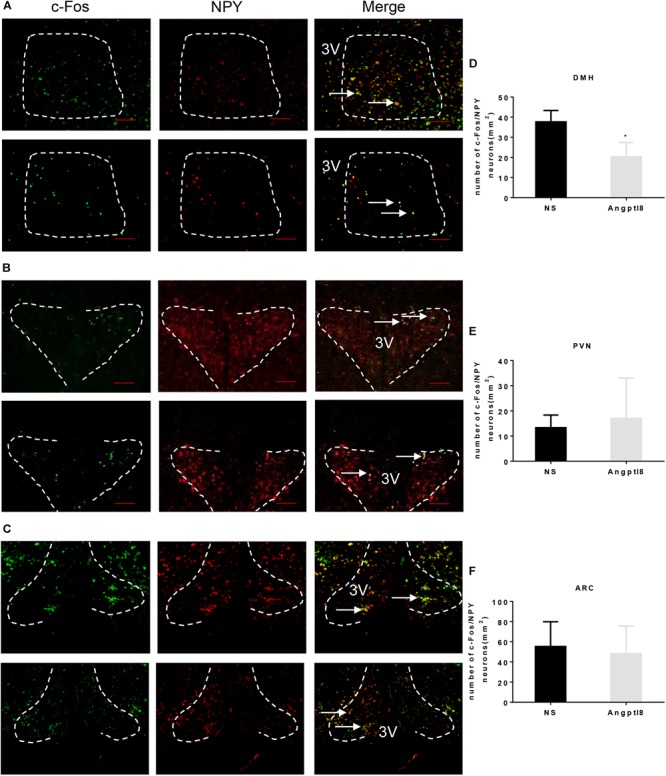
Double staining of c-Fos and NPY in the hypothalamic PVN, DMH, and ARC after the I.C.V. injection of Angptl8. **(A–C)** Double-immunofluorescence images showed c-Fos immunoreactive cells (green) and NPY neurons (red) separately, and neurons with colocalization in the PVN, DMH, and ARC are depicted in yellow in the merged photograph. Scale bars = 200 μm. **(D–F)** Colocalized neurons in the DMH (*n* = 4), PVN (*n* = 3), and ARC (*n* = 3) were counted and are expressed as means ± SEM. ^∗^*P* < 0.05 relative to the NS control group. Scale bar = 200 μm → means colocalization of c-Fos and NPY neurons.

A number of neurons associated with food intake are found in the hypothalamus. NPY neurons in the hypothalamus are involved in the development of appetite and mediate weight gain effects in the hypothalamus (Dube et al., 2007). Thus, we examined the colocalization of c-Fos/NPY neurons in the DMH (Figure 5A), PVN (Figure 5B), and ARC (Figure 5C) in which c-Fos immunoreactive neurons were altered by Angptl8 (Figure 4). Compared with the control group, Angptl8 (0.3 μg/μl) significantly decreased the coexpression of NPY- and c-Fos-positive neurons in the DMH (37.67 ± 5.69 vs. 20.25 ± 7.18 cells per section in NS and Angptl8 treated animals, respectively; *P* < 0.05; Figure 5D) but did not influence the merged area in the PVN (13.33 ± 5.03 vs. 17.00 ± 16.09 cells per section in NS and Angptl8 treated animals, respectively; *P* > 0.05; Figure 5E) or the ARC (55.33 ± 24.57 vs. 48.17 ± 27.46 cells per section in NS and Angptl8 treated animals, respectively; *P* > 0.05; Figure 5F). These data suggested that NPY-positive neurons in the DMH are involved in the anorexic effect of Angptl8.

### The Effect of Peripheral and Central Angptl8 on Body Weight Gain, Fat Mass, and Plasma Free Fatty Acid Levels

**FIGURE 6:**
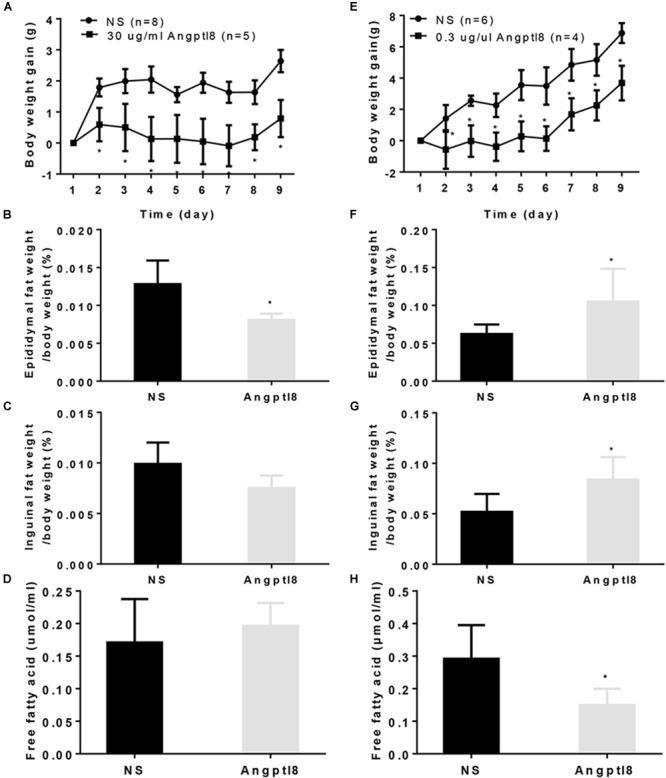
Effect of peripheral and central Angptl8 on body weight gain, fat mass and plasma FFA levels. Angptl8 (30 μg/ml) via I.V. injection decreased 9-day body weight gain **(A)** and epididymal fat deposits **(B)** but had no effect on inguinal fat deposits **(C)** or plasma free fatty acid levels **(D)**. Angptl8 (0.3 μg/μl) via I.C.V. injection decreased 9-day body weight gain **(E)** and plasma free fatty acid **(H)** but increased the epididymal **(F)** and inguinal **(G)** fat deposits. Data are means ± SEM. ^∗^*P* < 0.05 relative to the NS control group.

To determine whether chronic administration of Angptl8 causes any changes in long-term body weight gain, mice received I.V. injections of either Angptl8 (30 μg/ml) or NS once daily for 9 days. The 9-day body weight gain was substantially lower in the Angptl8 group than in the NS group (3.69 ± 1.10g vs. 6.88 ± 0.64g, *P* < 0.05; Figure 6A). The proportion of epididymal adipose tissue (0.01 ± 0.0008 g vs. 0.01 ± 0.0031 g, *P* < 0.05; Figure 6B) was lower than in the NS group; however, inguinal adipose tissue (0.01 ± 0.00 g vs. 0.01 ± 0.00 g, *P* > 0.05; Figure 6C) and plasma FFA levels (0.17 ± 0.07 g vs. 0.19 ± 0.03 g, *P* > 0.05; Figure 6D) were unchanged. In addition, 2 μl of Angptl8 at a concentration of 0.3 μg/μl was administered to the mice via I.C.V. injection for 9 days and 9-day body weight gain decreased significantly (0.79 ± 0.60g vs. 2.64 ± 0.36g, *P* < 0.05; Figure 6E). The proportions of epididymal adipose tissue (0.11 ± 0.04 g vs. 0.06 ± 0.01 g, *P* < 0.05; Figure 6F) and inguinal adipose tissue (0.08 ± 0.02 g vs. 0.05 ± 0.01 g, *P* < 0.05; Figure 6G) were greater in the Angptl8 group than in the NS group. However, the plasma FFA levels (0.15 ± 0.05 g vs. 0.10 ± 0.03 g, *P* < 0.05; Figure 6H) were lower in the Angptl8 group than in the NS group.

### Effect of Peripheral and Central Angptl8 on BAT Deposits, BAT Morphology and Ucp-1 Expression

**FIGURE 7:**
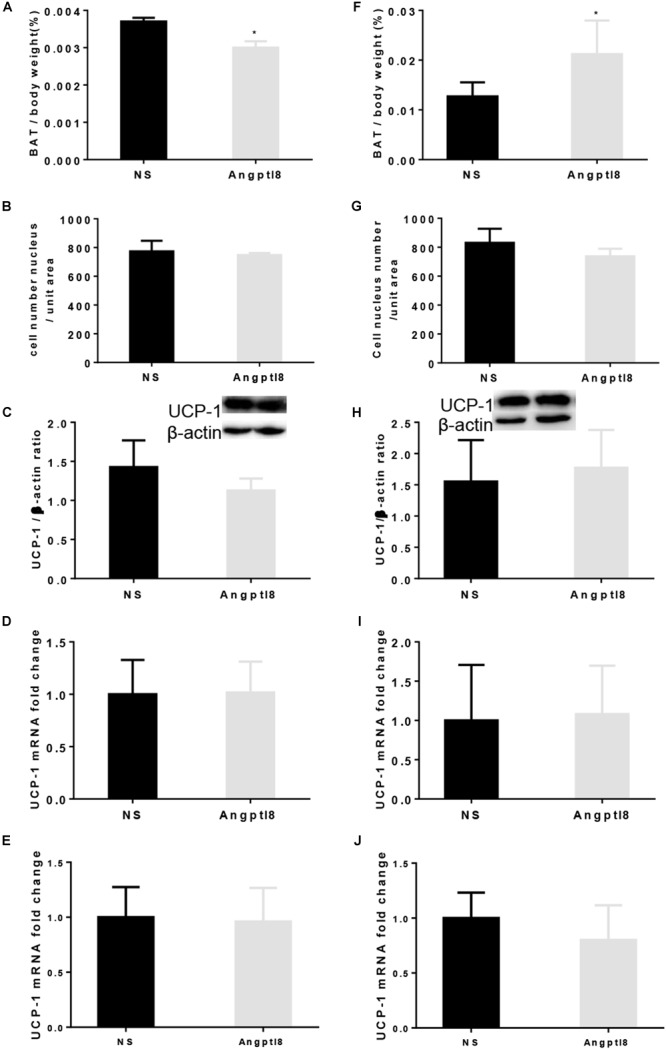
Effect of peripheral and central Angptl8 on BAT mass, BAT morphology, and Ucp-1 expression. Angptl8 (30 μg/ml) via I.V. injection (*n* = 6 and 3 in experimental and control group, respectively) decreased BAT deposit **(A)** but had no effect on BAT cell density **(B)** and Ucp-1 expression **(C–E)**. **(D)** Use β-actin as the HKG and **(E)** use GAPDH as the HKG. Angptl8 (0.3 μg/μl) via I.C.V. injection (*n* = 7 and 3 in experimental and control group, respectively) increased BAT deposit **(F)** but had no effect on BAT morphology **(G)** or Ucp-1 expression **(H–J)**. **(I)** Use β-actin as the HKG and **(J)** use GAPDH as the HKG. Data are means ± SEM. ^∗^*P* < 0.05 relative to the NS control group.

After the long-term I.V. administration of Angptl8 (30 μg/ml), the proportion of BAT (0.0030 ± 0.0001 g vs. 0.0037 ± 0.0030 g, *P* < 0.05; Figure 7A) was lower than that in the NS group. To further evaluate whether the weight loss caused by Angptl8 was related to the change in BAT in addition to the decreased adipose tissue deposits caused by decreased appetite, we measured the morphology of BAT and Ucp-1 expression. After I.V. injection, Angptl8 did not affect cell density in the BAT (773.58 ± 73.73 vs. 747.50 ± 14.55 cells/unit area, *P* < 0.05; Figure 7B) or Ucp-1 expression in the protein level (1.13 ± 0.15 vs. 1.43 ± 0.34 AU, *P* > 0.05; Figure 7C) or mRNA levels (1.02 ± 0.29 vs. 1.00 ± 0.33 AU, *P* > 0.05; Figure 7D and 0.96 ± 0.31 vs. 1.00 ± 0.27 AU, *P* > 0.05; Figure 7E). However, after the long-term I.C.V. injection of Angptl8 (0.3 μg/μl), the proportion of BAT (0.02 ± 0.01 g vs. 0.01 ± 0.00g, *P* < 0.05; Figure 7F was higher than that in the NS group. After the I.C.V. injection of Angptl8, the cell density of BAT (736.67 ± 53.15 vs. 830.71 ± 97.49 cells/unit area, *P* < 0.05; Figure 7G) and Ucp-1 protein (1.08 ± 0. 62 vs. 1.00 ± 1.00 AU, *P* > 0.05; Figure 7H) and mRNA levels (1.02 ± 0.29 vs. 1.00 ± 0.33 AU, *P* > 0.05; Figure 7I and 0.8 ± 0.32 vs. 1.00 ± 0.23 AU, *P* > 0.05; Figure 7J) were unchanged.

## Discussion

Although the roles of Angptl8 in inhibiting LPL activity and regulating serum triglyceride levels had been demonstrated (Zhang, 2012), the effect of exogenous Angptl8 on food intake is unclear. In the present study, we found that peripherally or centrally Angptl8 administration reduces cumulative food intake. The intravenous injection of Angptl8 slightly but significantly decreased food intake in a concentration-dependent manner (Figure 1A). An I.C.V. injection of high-concentration Angptl8 also significantly inhibited appetite in mice; a low concentration also inhibited appetite, but the difference between groups was not significant (Figure 1B), indicating that the higher concentration of Angptl8 should be used in further studies. These results are inconsistent with those of recent reports in which food intake was not affected by Angptl8 knockout (Banfi et al., 2018) or monoclonal antibody (REGN3776) treatment (Gusarova et al., 2017). The discrepancy may be explained by the different treatment modalities. Antibodies are typically too large to cross the blood-brain barrier (Gusarova et al., 2017); therefore, the monoclonal antibody may not exert an effect in the hypothalamus. In addition, we believe that Angptl8 is not an essential anorexic factor and the compensatory mechanisms plays crucial roles in energy homeostasis. Furthermore, the baseline food intake between experimental groups was quite different (6 g in Figure 1A and 8 g in Figure 1B), which may be attributed to the different kind of models.

Recently, Angptl8 has been detected in the brain (Quagliarini et al., 2012). In the present study, we found that Angptl8 is widely expressed in appetite-related nuclei, such as the DMH, PVN, VMH, and ARC (Figure 2). To evaluate whether Angptl8 exerts its function via the modulation of the activity of these appetite-related nuclei, c-Fos expression was measured after I.V. and I.C.V. Angptl8 administration. Surprisingly, both peripheral and central Angptl8 injection decreased the number of c-Fos immunopositive neurons in the DMH (Figures 3B, 4B). The DMH is a hypothalamic nucleus involved in energy homeostasis, including appetite, energy expenditure, and glucose homeostasis (Bi et al., 2012). Therefore, the DMH is an important mediator of the anorexia effects of exogenous Angptl8. Interestingly, peripheral or central Angptl8 administration influenced different nuclei in the hypothalamus. The peripheral injection of Angptl8 only increased c-Fos-immunopositive neurons in the DMH (Figure 3B). However, after central administration, the number of immunopositive neurons in the PVN (Figure 4C) and ARC (Figure 4D) were significantly affected, in addition to the DMH (Figure 4B). Although it is clear whether peripheral Angptl8 penetrates the blood-brain barrier, we inferred that peripheral and central Angptl8 both influence appetite-associated nuclei and influence energy homeostasis via different mechanisms.

Neuropeptide Y is an important orexigenic peptide expressed in appetite-related nuclei, such as the DMH (Clark et al., 1984). DMH lesions result in hypophagia (Bellinger and Bernardis, 2002) and the overexpression of NPY in the DMH of lean rats increases food intake (Yang et al., 2009). Moreover, NPY-immunopositive neurons are activated in the DMH during fasting (Palou et al., 2009) and in the obesity state (Bi et al., 2001). Immunofluorescence co-location of c-Fos and NPY were decreased in the DMH, suggesting that NPY-positive neurons in the DMH are involved in the anorexic effect of Angptl8. Generally, the activation of neurons expressing agouti-related protein (AgRP) and NPY strongly promotes feeding (Hahn et al., 1998; Boswell et al., 2002; Krashes et al., 2011; Kohno and Yada, 2012), while the activation of pro-opiomelanocortin (POMC) neurons activated by insulin or leptin has the opposite effects (Cone et al., 2001; Cowley et al., 2001). As Angptl8 increased c-Fos-immunopositive neurons in the ARC and decreased these neurons in the PVN, Angptl8 might influence POMC neurons and AgRP neurons in the ARC and PVN, respectively. Further studies are needed to clarify this phenomenon.

In addition to the acute experiments, a long-term study illustrated the chronic effects of Angptl8 on energy homeostasis. Peripheral (Figure 6A) and central (Figure 6E) Angptl8 both decreased body weight gain. In addition to feeding behavior, fat storage, and energy expenditure play essential roles in energy homeostasis. Surprisingly, peripheral Angptl8 injection decreased the visceral WAT mass (Figure 6B) in the chronic experiments, while the central injection increased fat deposits (Figures 6F,G) and decreased plasma FFA levels (Figure 6H). As Angptl8 inhibits the activity of LPL to decrease the plasma FFA levels and increases plasma triglyceride levels (Zhang, 2012; Wang et al., 2013; Haller et al., 2017), the increased WAT mass after central injection might due to the transformation of plasma FFA to triglycerides followed by deposition in adipose tissues. We conjecture that peripheral Angptl8 mainly affects appetite, but central Angptl8 has a more powerful effect on LPL activity.

As opposed to WAT, which functions in energy storage, BAT is involved in energy expenditure (Sell et al., 2004). A change in the interscapular BAT mass was in accordance with WAT after Angptl8 administration. Chronic exposure to Angptl8 increases the expression of brown adipocyte markers, such as Ucp-1, in adipose-derived stem cells (Martinez-Perez et al., 2016), implying that Angptl8 is involved in the regulation of browning, but Angptl8 did not enhance the transcription or translation of Ucp-1 in BAT in the present study. Although stem cells persist throughout life in many tissues, their properties shift over time to match the changing growth and regeneration demands of the tissues (Martinez-Perez et al., 2016). These results suggest the importance of further investigations of this system as well as its role in modulating fat mass and energy metabolism. Additional studies are needed to determine the other mechanisms by which Angptl8 regulates appetite and fat deposits.

## Conclusion

Angptl8 is involved in the energy homeostasis by inhibiting food intake via the DMH pathway in the DMH and by modulating the adipose tissue deposits.

## Author Contributions

JD, XC, and RW conceived and designed the experiments. RW, LW, YL, CZ, and LS performed the experiments. RW, JY, and XC analyzed the data. RW and JY wrote the paper. JD, XC, RW, and JY edited the manuscript. All authors read and approved the final manuscript.

## Conflict of Interest Statement

The authors declare that the research was conducted in the absence of any commercial or financial relationships that could be construed as a potential conflict of interest.

## Supplementary Material

FIGURE S1 The number of Angptl8 positive neurons in feeding and food deprivation state. Angptl8 was expressed in the hypothalamic DMH, PVN, VMH, and ARC in mice on chow diet and food deprivation for 12 h. ^∗^*P* < 0.05 relative to the feeding group.

## Abbreviations

Angptl8: angiopoietin-like protein 8
ARC: arcuate nucleus
BAT: brown adipose tissue
DMH: dorsomedial hypothalamus
FFA: free fatty acid
LPL: lipoprotein lipase
NPY: neuropeptide Y
PVN: paraventricular nucleus
Ucp-1: uncoupling protein 1
VMH: ventromedial hypothalamus
