# Supplementary figures and images for: Neuropeptide Y-Positive Neurons in the Dorsomedial Hypothalamus Are Involved in the Anorexic Effect of Angptl8
Source: Front Mol Neurosci. 2018 Dec 18;11:451. doi: 10.3389/fnmol.2018.00451 (PMC6305345; doi:10.3389/fnmol.2018.00451)

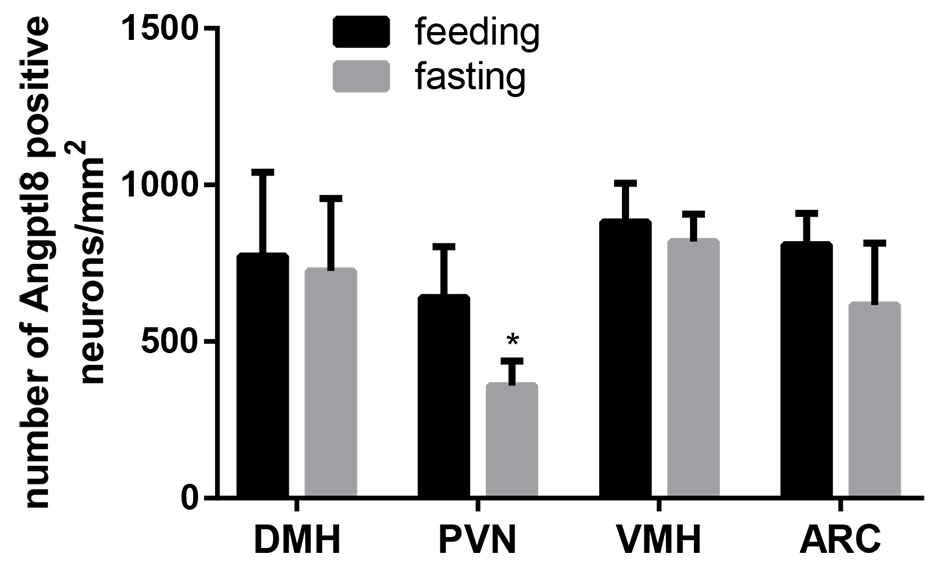

Supplement: FIGURE S1 — The number of Angptl8 positive neurons in feeding and food deprivation state. Angptl8 was expressed in the hypothalamic DMH, PVN, VMH, and ARC in mice on chow diet and food deprivation for 12 h. ∗P < 0.05 relative to the feeding group. [file Image_1.TIF]
